# LncRNA MALAT1 is dysregulated in diabetic nephropathy and involved in high glucose‐induced podocyte injury *via* its interplay with β‐catenin

**DOI:** 10.1111/jcmm.13189

**Published:** 2017-04-26

**Authors:** Mengsi Hu, Rong Wang, Xiaobing Li, Minghua Fan, Jiangong Lin, Junhui Zhen, Liqun Chen, Zhimei Lv

**Affiliations:** ^1^ Department of Nephrology Shandong Provincial Hospital Affiliated to Shandong University Jinan China; ^2^ Institute of Basic Medicine Shandong Academy of Medical Sciences Jinan China; ^3^ Department of Obstetrics and Gynecology The Second Hospital of Shandong University Jinan China; ^4^ Department of Pathology School of Medicine Shandong University Jinan China; ^5^ Department of Nephrology First Affiliated Hospital of Chongqing Medical University Chongqing China

**Keywords:** MALAT1, β‐catenin, SRSF1, podocyte, diabetic nephropathy, high glucose

## Abstract

Metastasis associated lung adenocarcinoma transcript 1(MALAT1) is a long non‐coding RNA, broadly expressed in mammalian tissues including kidney and up‐regulated in a variety of cancer cells. To date, its functions in podocytes are largely unknown. β‐catenin is a key mediator in the canonical and non‐canonical Wnt signalling pathway; its aberrant expression promotes podocyte malfunction and albuminuria, and contributes to kidney fibrosis. In this study, we found that MALAT1 levels were increased in kidney cortices from C57BL/6 mice with streptozocin (STZ)‐induced diabetic nephropathy, and dynamically regulated in cultured mouse podocytes stimulated with high glucose, which showed a trend from rise to decline. The decline of MALAT1 levels was accompanied with β‐catenin translocation to the nuclei and enhanced expression of serine/arginine splicing factor 1 (SRSF1), a MALAT1 RNA‐binding protein. Further we showed early interference with MALAT1 siRNA partially restored podocytes function and prohibited β‐catenin nuclear accumulation and SRSF1 overexpression. Intriguingly, we showed that β‐catenin was involved in MALAT1 transcription by binding to the promotor region of MALAT1; β‐catenin knock‐down also decreased MALAT1 levels, suggesting a novel feedback regulation between MALAT1 and β‐catenin. Notably, β‐catenin deletion had limited effects on SRSF1 expression, demonstrating β‐catenin might serve as a downstream signal of SRSF1. These findings provided evidence for a pivotal role of MALAT1 in diabetic nephropathy and high glucose‐induced podocyte damage.

## Introduction

Long noncoding RNAs (lncRNAs) are non‐protein‐coding transcripts longer than 200 nucleotides and have emerged as important regulators in a variety of cellular responses, developmental and disease processes 1. Evidence has shown that lncRNAs are associated with TGF‐β/Smad3‐mediated renal inflammation and fibrosis 2 and might be functionally important in modulating renal responses to hyperglycaemia and the progression of diabetic nephropathy, a major microvascular complication of diabetes and one of the leading causes of end‐stage kidney diseases 3. Podocytes are a key component of kidney filtration barrier 4, 5. Loss of podocytes has been indicated as an important early pathologic marker of diabetic nephropathy 6. Recent studies demonstrated MALAT1, one of the first discovered lncRNAs, was significantly up‐regulated in retinal endothelial cells under culture of high glucose and in the retinas of diabetic mice 7, 8, 9, implying a potential involvement of MALAT1 in diabetic microvascular complications. Therefore, it is of great interest to identify whether MALAT1 plays a role in diabetic nephropathy and high glucose‐associated podocyte damage.

Prior studies showed MALAT1 acted as an important oncogene in numerous cancers 10, 11. It promoted cell migration and invasion *via* epithelial‐mesenchymal transition (EMT) mechanism in oral squamous cell carcinoma (OSCC), when both NF‐κB and β‐catenin were triggered nuclear translocation 12. β‐catenin is a key intracellular signal transducer in the Wnt signalling pathway 13, which is hyperactivated in response to injury and contributes to kidney fibrosis 14. Its excessive activation is also found in glomeruli of focal segmental glomerulosclerosis and diabetic nephropathy 15, 16. When activated canonical Wnt signalling or other signalling pathways converges at β‐catenin, it enters the nucleus to modulate expression of EMT regulatory genes such as Twist, Snail and Slug 17. In our previous studies, we showed podocytes underwent EMT after high glucose treatment when aberrant expression of Snail and dephosphorylated β‐catenin were observed 18. However, little information is available to date illustrating whether there is any interaction between MALAT1 and β‐catenin in podocytes.

Mature MALAT1 is localized in the cell nuclei with a cytoplasmic tRNA‐like small RNA, known as mascRNA 19. When RNA polymerase II‐dependent transcription is active, MALAT1 becomes enriched in nuclear speckles‐dynamic and irregularly shaped nuclear domains involved in pre‐mRNA splicing processing and RNA transport in mammalian cells 20, 21. SRSF1 (also known as SF2/ASF) belongs to the SR family, one of the major pre‐mRNA splicing factors; in addition to its pre‐mRNA splicing abilities, SRSF1 is also involved in other cellular steps of RNA metabolism, such as nuclear export of the mature mRNA, nonsense‐mediated mRNA decay (NMD) and mTOR activation by shuttling between the nucleus and the cytoplasm 22, 23. Recent studies suggested that human SRSF1 and SRSF9 were able to enhance Wnt1 as well as β‐catenin‐induced reporter expression, whereas SRSF2 could not 24. It was found that MALAT1 promoted cell proliferation in gastric cancer by recruiting SRSF1 and modulated SRSF1 distribution to nuclear speckles in Hela cells 25, 26. Of note, in WT1 mutant cells, serine/arginine protein kinase 1 (SRPK1)‐mediated hyperphosphorylation of SRSF1 promoted the expression of pro‐angiogenic VEGF‐a splice isoforms and caused imbalanced angiogenesis, a prerequisite for tumour growth 27 and diabetic nephropathy 28. These observations prompt us to investigate the potential functions of SRSF1, and its interplay with MALAT1 lncRNA in mouse podocytes.

In this study, we provided evidence that MALAT1 lncRNA was dysregulated in STZ‐induced diabetic nephropathy when proteinuria was marked and involved in high glucose‐induced podocyte damage. MALAT1 knock‐down rectified podocyte damage *via* down‐regulating SRSF1 overexpression, a MALAT1 lncRNA‐binding protein, and partial reversal of β‐catenin nuclear accumulation triggered by high glucose. In addition, we showed MALAT1 levels were also under the regulation of β‐catenin *via* its binding ability to MALAT1 promotor region; β‐catenin gene knock‐down led to decreased MALAT1 levels, demonstrating a novel feedback regulation between MALAT1 and β‐catenin.

## Materials and methods

### Reagents

Antibodies specific for β‐catenin and SRSF1 were purchased from Abcam (Cambridge, MA, USA). Anti‐p‐cadherin antibodies purchased from Santa Cruz Biotechnology, Inc. (Santa Cruz, CA, USA). Anti‐ZO‐1 antibodies were purchased from Thermo Fisher Scientific Inc. (Rockford, IL, USA). Anti‐desmin antibodies were from Immunoway. Anti‐β‐actin antibodies were obtained from Proteintech (Chicago, IL, USA). Secondary antibodies were from Jackson ImmunoResearch Laboratories, Inc. (West Grove, PA, USA). Dylight 594 and 488 conjugated secondary antibodies were from Abcam (Burlingame, CA, USA).

### Animal studies

A total of 20 male C57BL/6 mice were purchased from the Animal Center Affiliated to Shandong University. All animal studies were carried out with the review and approval of the animal care and use committee of Shandong University. Mice were randomly allocated into two groups, either intraperitoneal injected with STZ dissolved in a low pH (pH 4.5) citrate buffer (50 mg/kg for 5 consecutive days) or citrate buffer alone. Hyperglycaemic state was monitored and maintained on a standard rodent diet with water *ad libitum*; urine was collected for the determination of urinary protein when the mice were weighed and placed in metabolic cages for 24 hrs. At the end of 12 weeks after the onset of diabetes, mice were killed, and the kidneys were harvested for the following experiments.

### Cell culture

Conditionally immortalized mouse podocytes were kindly provided by Professor Peter Mundel (Massachusetts General Hospital, Boston, MA, USA) *via* Professor Jie Ding (Peking University, Beijing, China). In brief, podocytes were cultured on type I collagen in RPMI 1640 supplemented with 10% foetal bovine serum (FBS) (Life Technologies Corporation, CA, USA), 100 U/ml penicillin and 100 mg/ml streptomycin under permissive conditions 33°C plus 10 U/ml mouse recombinant γ‐interferon (Pepro Technology, Rocky Hill, NJ, USA). Cell differentiation was induced by maintaining podocytes on type I collagen at 37°C without γ‐interferon under non‐permissive conditions for at least 14 days 18, 29. HEK‐293 cells were cultured in Dulbecco's Modified Eagle's Medium (DMEM) supplemented with 10% FBS.

### siRNA transfection and lentiviral vector transduction

MALAT1 siRNAs (sense 5′‐GCCUUGUAGAUUAAAACGAtt‐3′, antisense 5′‐UCGUUUUAAUCUACAAGGCcg‐3′) and a negative control sequence (control siRNA) were purchased from Invitrogen (Thermo Fisher Scientific Inc.). Transfection of podocytes was performed (0.5 × 10^5^ cells/well in a 24‐well plate) with the indicated siRNAs using Lipofectamine^®^ RNAiMAX Transfection reagent (Thermo Fisher Scientific Inc.). Real‐time reverse transcriptase‐PCR was used to validate the efficiency of MALAT1 knock‐down. After a 48‐hrs incubation with MALAT1 siRNA or control siRNA, podocytes were then treated with high glucose (30 mM) for an additional 48 hrs. Cells that were not transfected and incubated with low‐glucose (5.6 mM) medium for 48 hrs were the controls. The lentiviral shRNA constructs targeting mouse β‐catenin (5′‐GCTGCGGAAGATGGGATCAAA‐3′) and a scrambled shRNA (5′‐GACTTCATAAGGCGCATGC‐3′), as well as lentiviral expression vectors for murine β‐catenin, and empty control vectors were purchased from Cyagen (Guangzhou, China), and lentivirus infection of the vectors was performed following the protocol provided by the manufacturer. Briefly, viral supernatants were added for 24 hrs to infect mouse podocytes. And geneticin (250 mg/ml) was added into the culture medium to select cells with stable viral integration.

### Real‐time reverse transcriptase‐PCR

Real‐time reverse transcriptase‐PCR was used to detect the gene expression in cultured podocytes and mouse kidney cortices. Extraction and concentration calculation of total RNA were described as previously 18. Aliquots of total RNA (1.0 μg each) from each sample were reverse transcribed into cDNA according to the instructions of PrimeScript^®^ RT Reagent Kit (Takara, Dalian, China). Briefly, after reverse transcription of total RNA, cDNA was used as a template for the PCR reactions using gene‐specific primer pairs. Amplification was performed using SYBR^®^ Premix Ex Taq™ Kit (Takara, Dalian, China) in the LightCycler^®^ 480 Real‐Time PCR system (Roche Applied Science, F. Hoffmann‐La Roche Ltd, Pleasanton, CA, USA). The primers were purchased from Sangon Biotech Co., Ltd (Shanghai, China). The sequences were designed as follows (Table 1):

**Table 1:** Primers for real‐time PCR

Gene	Primer	Sequence
P‐cadherin	Sense	5′‐GTTTGAGCCGCAGAAGTATGA‐3′
	Antisense	5′‐GAGTGGTGATGGTGAAATGGT‐3′
ZO‐1	Sense	5′‐GAGCTACGCTTGCCACACTGT‐3′
	Antisense	5′‐TCGGATCTCCAGGAAGACACTT‐3′
Desmin	Sense	5′‐GCAGCCAATAAGAACAACGAT‐3′
	Antisense	5′‐ATCAATCTCGCAGGTGTAGGA‐3′
β‐catenin	Sense	5′‐CCATCACGACACTGCATAATCT‐3′
	Antisense	5′‐AGCCAAGAATTTCACGTTTGTT‐3′
SRSF1	Sense	5′‐TATCCGAACCAAGGACATCG‐3′
	Antisense	5′‐AACTCAACGAAGGCGAAGG‐3′
MALAT1	Sense	5′‐GTTACCAGCCCAAACCTCAA‐3′
	Antisense	5′‐CGATGTGGCAGAGAAATCAC‐3′
β‐actin	Sense	5′‐AAGACGAGGAGGAACTGAAC‐3′
	Antisense	5′‐CAAATCGGA CAACAAGACG‐3′
MALAT1‐RIP‐1 (Mouse)	Sense	5′‐CCATTTTCAGTGTGGGGATT‐3′
	Antisense	5′‐TCGTTCACCTGTTGTCCTCA‐3′
MALAT1‐RIP‐2 (Mouse)	Sense	5′‐CACCAGAAAAGGCCATCAAT‐3′
	Antisense	5′‐CTACATTCCCACCCAGCACT‐3′
MALAT1‐ChIP (Mouse)	Sense	5′‐TGGTCCTGGGAGAAAGAGAA‐3′
	Antisense	5′‐CTGCGCTAATAACCGTCAGAG‐3′
MALAT1‐RIP‐1 (Human)	Sense	5′‐ATGCGAGTTGTTCTCCGTCT‐3′
	Antisense	5′‐CTTATCTGCGGTTTCCTCAAG‐3′
MALAT1‐RIP‐2 (Human)	Sense	5′‐AGTTCAGTGTTGGGGCAATC‐3′
	Antisense	5′‐ACATTCGTTCTTCCGCTCA‐3′
MALAT1‐ChIP (Human)	Sense	5′‐GCCATTTTAGCAACGCAGA‐3′
	Antisense	5′‐CAAGGACTCTGGGAAACCTG‐3′

### Western blot analysis

The total protein was extracted from podocytes under different conditions with ice‐cold lysis buffer containing proteinase inhibitors and phosphatase inhibitors. About 50 μg of total protein was separated by SDS‐PAGE and transferred to polyvinylidene fluoride (PVDF) membranes; the membranes were then blocked with 5% milk or BSA for 1 hr and incubated at 4°C overnight with primary antibodies against the following target proteins: ZO‐1 (1:1000), p‐cadherin (1:1000), desmin (1:1500), β‐catenin (1:1000), SRSF1 (1:1000) and β‐actin (1:1000). The membranes were then washed three times with TBST for 5 min. and incubated with species‐specific peroxidase‐conjugated secondary antibodies diluted in blocking buffer for 1 hr at room temperature. Specific bands were detected using the ECL system and the Bio‐Rad electrophoresis image analyser (Bio‐Rad, Hercules, CA, USA).

### Gelatin zymography

MMP‐2 enzyme activity of the podocytes under different conditions was monitored by gelatin zymography. Briefly, supernatants from stimulated or control cells were collected and centrifuged and were mixed with 4 × non‐reducing loading buffer (Solarbio, Beijing, China). Protein separation was performed by electrophoresis using 10% polyacrylamide SDS gel containing 1 mg/ml gelatin, after which the gels were denatured in 2.5% Triton X‐100 for 45 min., equilibrated in buffer (50 mM Tris‐HCl, 5 mM CaCl_2_ and 1 μM ZnCl_2_ [pH 7.6]) for 40 min. followed by incubation in developing buffer (50 mM Tris‐HCl, 5 mM CaCl_2_, 1 μM ZnCl_2,_ and 0.02% Brij‐35 [pH 7.6]) at 37°C for 36 hrs. Gels were stained with 0.05% bright blue R‐250 for 3 hrs, and then washed with distaining solution (methanol: acetic acid: water 30:10:60) and quantified using NIH image.

### Immunofluorescence

The mouse kidneys were paraffin embedded, sectioned at 4 μm, and subjected to antigen retrieval followed by blocking with 1% BSA for 1 hr at room temperature to block non‐specific binding. Cultured podocytes under different conditions were plated onto different 6‐well plates and fixed in 4% paraformaldehyde for 10 min., followed by 1% BSA (with 0.3% Triton X‐100) for 1 hr at room temperature to block non‐specific binding. Immunostaining was performed with appropriate primary antibodies at 4°C overnight and Dylight 594 or 488‐conjugated IgG at room temperature for 1 hr for visualization. 4′, 6‐diamidino‐2‐phenylindole (DAPI) was used to visualize the nuclei. Images were observed and captured on an inverted phase/fluorescence microscope (Leica Microsystems GmbH, Wetzlar, Germany).

### Fluorescence *in situ* hybridization

MALAT1 probe and fluorescence *in Situ* Hybridization kit were purchased from Ribio (Guangzhou, China). Briefly, cultured podocytes under different conditions were plated onto different 6‐well plates and fixed in 4% paraformaldehyde for 10 min., followed by ice‐cold PBS containing 0.5% Triton X‐100 for 5 min., and then incubated with pre‐hybridization buffer for 30 min. at 37°C to block non‐specific binding. Hybridization buffer was preheated in a 37°C water bath, and the MALAT1 FISH probe working buffer was prepared by diluting probe stock solution in hybridization buffer (1:40). Cells were incubated with probe working buffer at 37°C overnight and followed by DAPI staining to visualize the nuclei. Images were observed and captured on an inverted phase/fluorescence microscope (Leica Microsystems GmbH, Wetzlar, Germany).

### RNA‐binding protein immunoprecipitation

RNA‐binding protein immunoprecipitation was performed using Magna RIP kit (#17‐10521; Millipore, Billerica, MA, USA). In brief, podocytes or HEK‐293 cells were fixed by 0.3% formaldehyde and quenched with glycine, and scraped to pellet cells, which were re‐suspended and collected by high speed vortex and spinning at 800 *g* at 4°C for 5 min. For sonication, nuclei pellets were re‐suspended in 500 μl RIP Cross‐linked Lysis Buffer containing protease/RNase inhibitors and sonicated on wet ice using a sonicator (VCX 455, Sonics & Materials Inc, Newton, CT, USA). Antibody‐beads complex were prepared, and 50 μl sheared cross‐linked chromatin was incubated with antibody‐beads with rotation overnight at 4°C. Cross‐link reversal was performed by incubating the beads in 200 μl elution buffer containing 10% SDS and proteinase K with shaking at 60°C for 30 min. RNA extraction was described previously 18. Aliquots of total RNA (150 ng each) from each sample were reverse transcribed into cDNA, and the enrichment of RNA was determined by real‐time PCR.

### Chromatin immunoprecipitation assay

According to the EZ‐Magna CHIP™ HiSens kit (#17‐10461; Millipore, Billerica, MA, USA), 1 × 10^7^ podocytes with or without catenin gene knock‐down or HEK‐293 cells were fixed with 1% PFA, quenched with glycine, washed with cold PBS and re‐suspended in nuclei isolation buffer with a protease inhibitor cocktail. The chromatin was sonicated on wet ice to obtain chromatin fragment lengths between 200 and 1200 bp. The protein‐DNA complexes were incubated with 1 μg of anti‐β‐catenin, or non‐specific IgG antibodies couple with protein A/G beads overnight followed by washing and elution. Cross‐links were reversed at 65°C for 2 hrs and 95°C for 15 min. DNA was purified and subjected (20 ng each) to real‐time PCR analysis.

### Dual‐luciferase reporter assay

The luciferase reporter constructs containing the MALAT1 promoter region were generated by PCR using the primers: sense 5′‐TGGTCCTGGGAGAAAGAGAA‐3′; antisense 5′‐CCTGGGAGGACAGAGGGTAT‐3′. Briefly, cultured podocytes stably overexpressing β‐catenin or control cells were cotransfected with pGL3‐*MALAT1 promotor*‐luciferase reporter vectors and pGL3‐TK‐Renilla luciferase control vectors for 24 hrs using Lipofectamine 3000 (Thermo Fisher Scientific Inc.) with or without further incubation with either low (5.6 mM) or high‐glucose (30 mM) treatment for 48 hrs. The luciferase activities were analysed using a Dual‐Luciferase^®^ Reporter Kit (Promega, Madison, WI, USA).

### Podocyte permeability assay

To determine the permeability of albumin influx, differentiated podocytes were seeded on collagen I‐coated Transwell filters (Corning One Riverfront Plazza, NY, USA) and stimulated either with low glucose or high glucose. After 48 hrs of stimulation, the top chamber was refilled with 200 μl of RPMI 1640 media and the bottom with 600 μl of RPMI 1640 media containing 1.5 mg/ml FITC‐albumin (Solarbio). Cells were then incubated at 37°C, and 30 μl of media from the top chamber were carefully collected at the indicated time‐points. The absorbance of FITC‐albumin was determined at 490 nm with a fluorescence multi‐well plate reader (SpectraMax M3, Molecular Devices, Sunnyvale, CA, USA) to indicate the filtration/influx function of monolayer podocytes.

### Statistical analysis

Experiments were performed at least three times. Values were reported as mean ± S.D. Data were analysed using SPSS 19.0 software. Statistical significance was assessed using Student's *t*‐test, one‐way anova and LSD‐*t* test, and two‐way anova. *P* < 0.05 were considered to be statistically significant.

## Results

### LncRNA MALAT1 was up‐regulated in the kidney of STZ‐induced diabetic mice

Previous studies demonstrated MALAT1 was significantly increased in high glucose‐treated retinal endothelial cells and in the retinas of diabetic mice 9, implying a role of MALAT1 in diabetes microvascular complication. In this study, we established a mice model of diabetic nephropathy by STZ injection and assessed the levels of MALAT1 in kidney samples. Results revealed that by 12 weeks after the onset of diabetes, diabetic mice weighed significantly less with their blood glucose maintained at high levels, in comparison with the control (*P* < 0.01) (Table 2).

**Table 2:** Metabolic data at 12 weeks of experiment

Groups	Control	Diabetic mice
Blood glucose (mg/dl)	6.44 ± 0.89	27.037 ± 2.78*
Body weight (g)	31.7 ± 1.9	23. 2 ± 1.6*
UTP (μg/24 hrs)	27.71 ± 9.34	123.43 ± 24.17*

Data are mean ± S.D.; *n* = 10 in each group; *P < 0.01 *versus* control.

**Figure 1:**
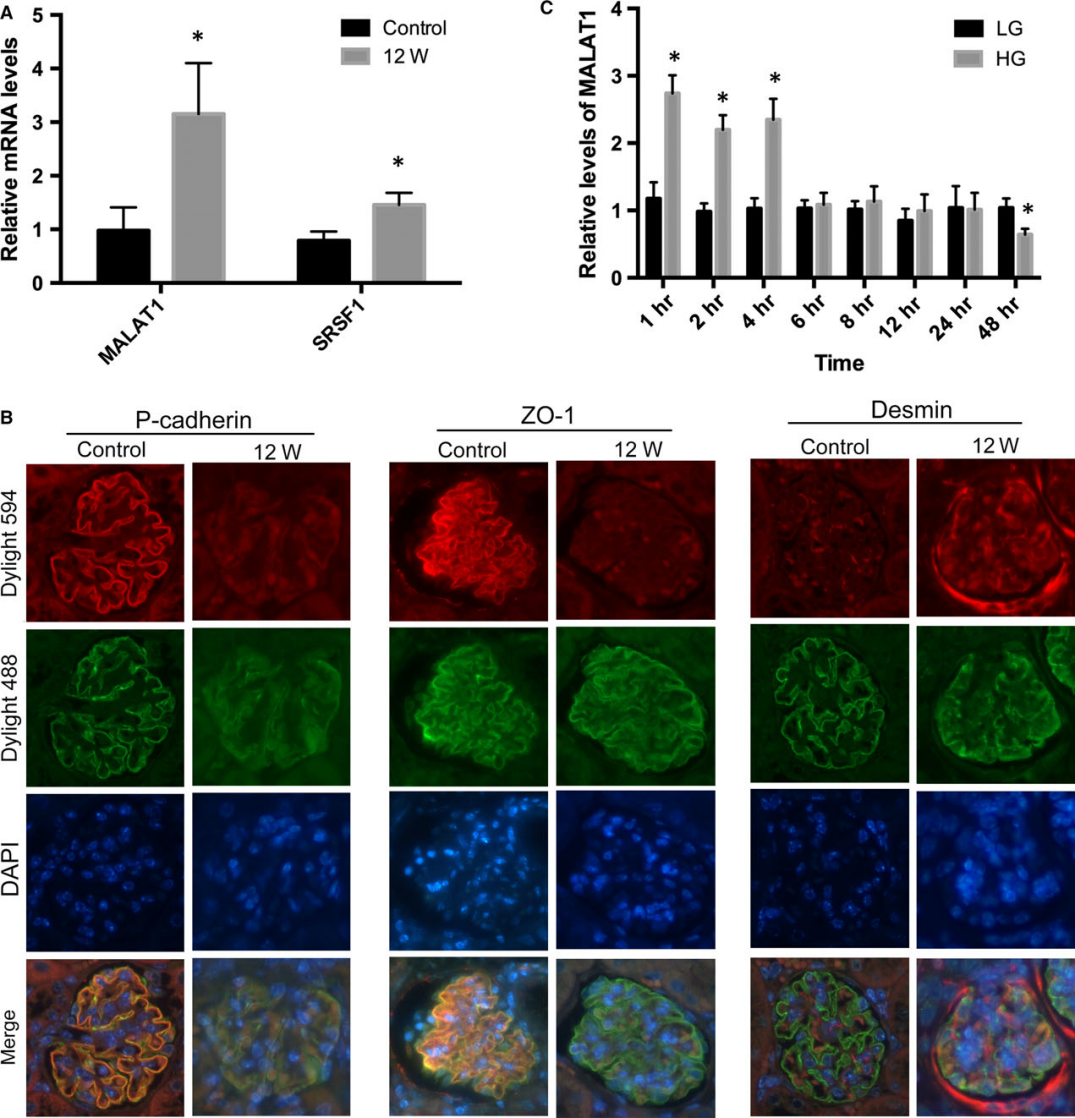
STZ‐induced diabetic podocyte injury and MALAT1 alterations *in vivo* and *in vitro*. (**A**) PCR analysis showed MALAT1 and SRSF1 were significantly up‐regulated in the kidney at 12 weeks after the onset of diabetes, in comparison with the control. Values denote the mean ± S.D.; *P < 0.01 *versus* control. (**B**) Immunofluorescence presented that in the control mice, p‐cadherin and ZO‐1 were intensely expressed in a leaner setting along the glomeruli, whereas markedly blunted in the kidney after STZ injection; on the contrary, desmin expression was hardly determined in the control but highly expressed in diseased glomerular podocytes. Synaptopodin was double stained to define the edge of glomerular podocytes. Magnification 400 × . (**C**) In cultured mouse podocytes, high‐glucose treatment (HG) (30 mM) resulted to a remarkable up‐regulation of MALAT1 at time‐points of 1, 2 and 4 hrs, and a reduction afterwards to a level comparable to podocytes in exposure to low‐glucose (LG) (5.6 mM) conditions. HG stimulation for 48 hrs led to a marked decline in MALAT1 expression, compared with podocytes treated with LG. Values denote the mean ± S.D.; *P < 0.01 *versus* LG.

Concomitantly, MALAT1 levels were remarkably up‐regulated in the kidney cortices of diabetic mice, compared with that of non‐diabetic mice (*P* < 0.01) (Fig. 1A), with a radical impairment of glomerular podocytes and marked proteinuria (*P* < 0.01) (Table 2). Immunofluorescence showed that in the glomeruli of control mice p‐cadherin and ZO‐1 were highly expressed in a linear pattern, but were both significantly reduced at 12 weeks; the staining of desmin, on the contrary, was enhanced at 12 weeks, whereas in normal glomerular podocytes its expression was hardly detected (Fig. 1B). P‐cadherin was expressed in a pattern comparable to that of ZO‐1 at cell‐cell contacts in both undifferentiated and differentiated podocytes 18. Together with nephrin, p‐cadherin was reported as an important component of SD in podocytes, necessary for the maintenance of the three‐dimensional structure of podocytes 30, 31, 32. Desmin was one of the intermediate filament proteins and has been widely recognized as an important indicator for podocyte injury 33. These observations demonstrated that MALAT1 dysregulation was associated with the development of proteinuria and progression of diabetic nephropathy. In addition, we showed mRNA levels of SRSF1, one of the major pre‐mRNA splicing factors, were upmodulated at 12 weeks (*P* < 0.01) (Fig. 1A), suggesting SRSF1 might also have an engagement in diabetic nephropathy.

### MALAT1 levels in cultured podocytes were dynamically regulated under high‐glucose conditions

**Figure 2:**
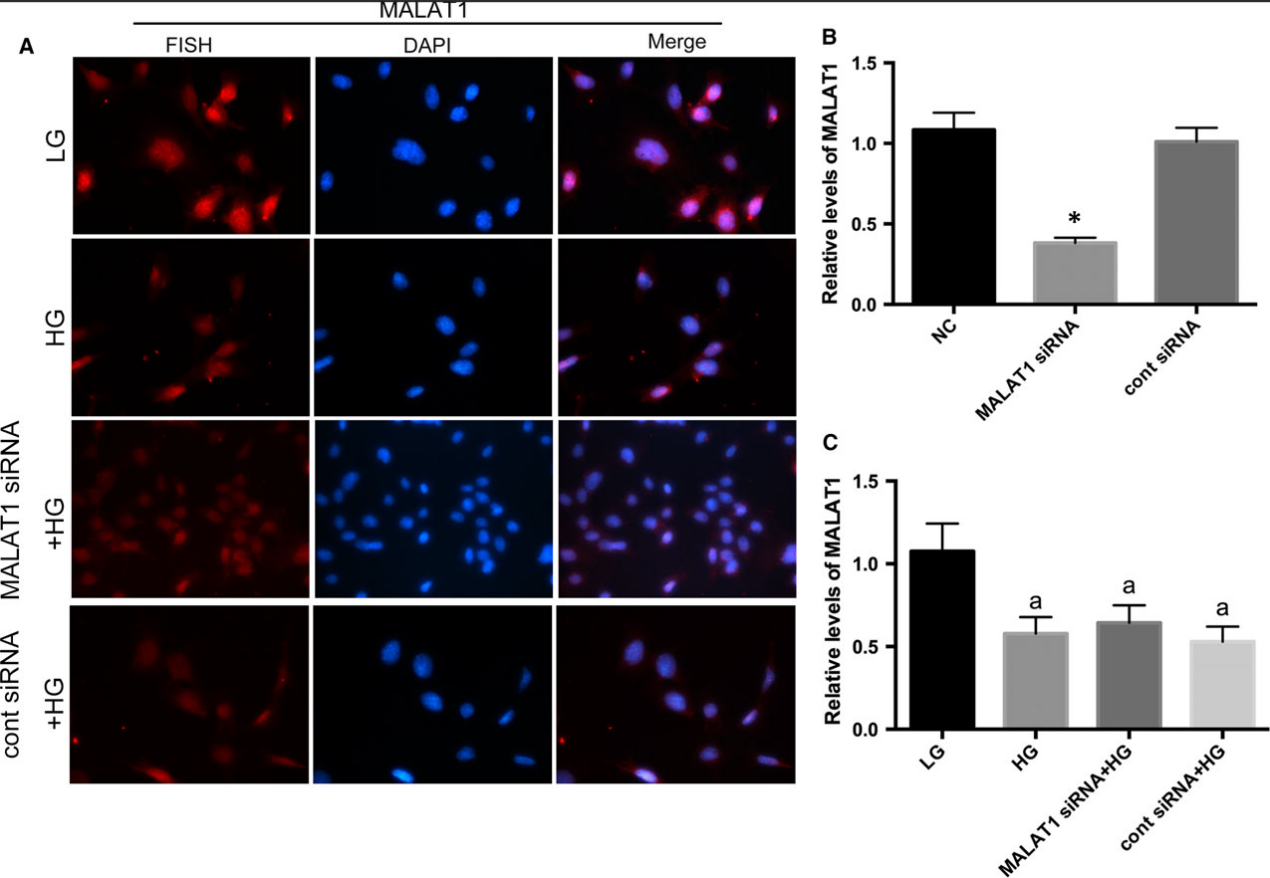
Determination of MALAT1 location mouse podocytes under culture. (**A**) By FISH, we observed a nuclear location of MALAT1 in cultured mouse podocytes. HG for 48 hrs significantly decreased MALAT1 staining, without resulting in any dislocation of MALAT1; however, MALAT1 siRNA transfection did not lead to a further extent of obliteration of MALAT1 staining, compared with podocytes without transfection. Podocytes under culture of LG or transfected with cont siRNA were the controls. (**B**) MALAT1 knock‐down efficiency was examined by real‐time PCR, which showed a significant abrogation of MALAT1 after transfection of MALAT1 siRNA for 48 hrs. Podocytes under normal conditions or transfected with cont siRNA were the controls. *P < 0.01 *versus* NC or cont siRNA. (**C**) Real‐time PCR analysis showed that HG treatment for 48 hrs resulted a remarkable reduction in MALAT1 levels; MALAT1 siRNA transfection did not lead to a further extent of obliteration of MALAT1 staining, compared with podocytes without transfection. Podocytes under culture of LG or transfected with cont siRNA were the controls. Values denote the mean ± S.D.; ^a^*P* < 0.01 *versus* LG.

To date, little information is known about the functions of MALAT1 in glomerular podocytes, a pivotal component of kidney filtration barrier. In this study, MALAT1 expression was determined in cultured mouse podocytes treated with high glucose. It was found by real‐time PCR that high‐glucose stimulation led to a twofold increase in MALAT1 levels at the time‐points of 1, 2, 4 hrs (*P* < 0.01), after which MALAT1 was restored to a level comparable to that in podocytes under low‐glucose conditions (*P* > 0.05) but notably decreased until 48 hrs of treatment (*P* < 0.01), showing a trend from rise to decline (Figs 1C and 2C). FISH revealed that MALAT1 was localized in the nuclei of normal cultured mouse podocytes; 48 hrs of high glucose was sufficient to weaken the *in situ* signals without interfering the nuclear location of MALAT1 (Fig. 2A).

**Figure 3:**
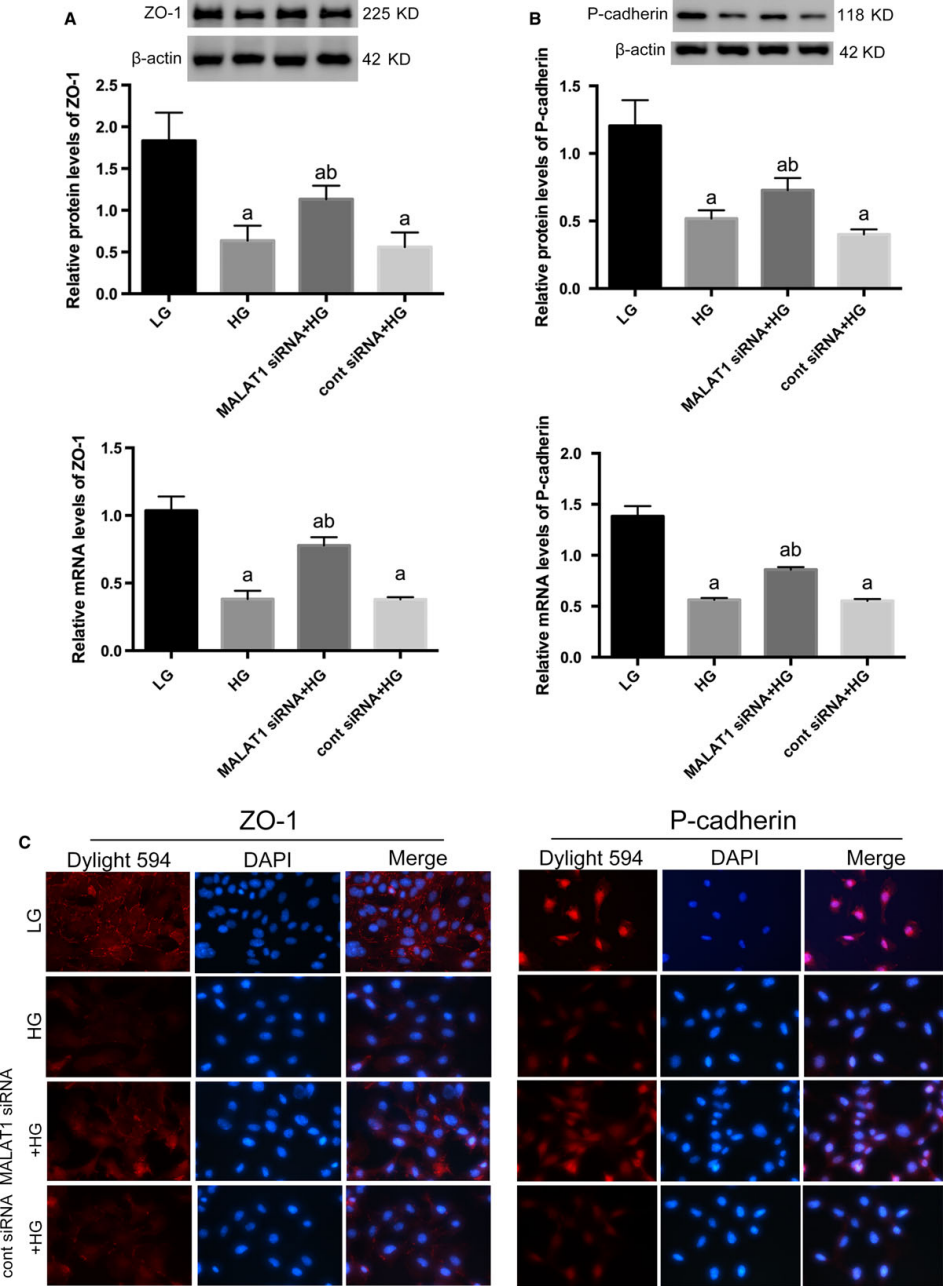
Determination of cellular markers of podocytes. (**A,B**) Western blot and real‐time PCR showed specific markers ZO‐1 and p‐cadherin were abundantly expressed in podocytes under LG conditions, both of which were significantly prohibited by HG on both protein and mRNA levels. And these abnormalities were abrogated by MALAT1 knock‐down. Podocytes under culture of LG or transfected with cont siRNA were the controls. Values denote the mean ± S.D.; ^a^*P* < 0.01 *versus* LG,^b^*P* < 0.05 *versus* HG. (**C**) Immunofluorescence showed a 48‐hrs incubation with HG led to decreased staining of p‐cadherin and ZO‐1, in comparison with cells under LG conditions when ZO‐1 was intensely located at cell‐cell contacts and p‐cadherin highly expressed at cytoplasm and nuclei; these alterations were partially rectified by MALAT1 siRNA transfection, presenting early prohibition of MALAT1 was protective for HG‐treated podocytes. Magnification 400 × .

**Figure 4:**
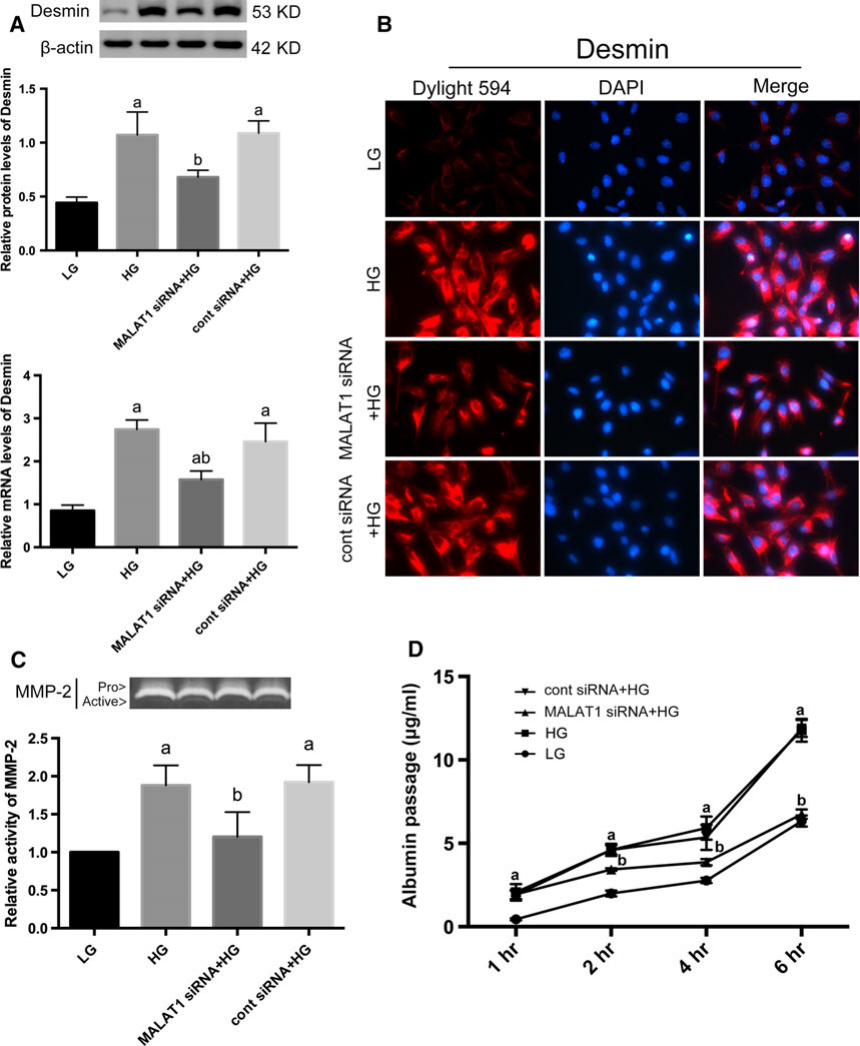
Determination of injury markers of podocytes. (**A**) By Western blot and real‐time PCR analysis, we showed desmin expression was induced by HG stimulation on both protein and mRNA levels, whereas diminished by MALAT1 siRNA transfection. Podocytes under culture of LG or transfected with cont siRNA were the controls. Values denote the mean ± S.D.; ^a^*P* < 0.01 *versus* LG,^b^*P* < 0.05 *versus* HG. (**B**) Immunofluorescence further confirmed the cellular damage and restoration of podocytes. Desmin was faintly stained under LG conditions but highly expressed in a paranuclear pattern after HG incubation for 48 hrs; MALAT1 siRNA transfection prevented and reduced staining of desmin. Magnification 400 × . (**C**) Gelatin zymography demonstrated a hyperactivity of MMP‐2 after HG treatment for 48 hrs, which was ameliorated by MALAT1 siRNA transfection. Podocytes under culture of LG or transfected with cont siRNA were the controls. Values denote the mean ± S.D.; ^a^*P* < 0.01 *versus* LG,^b^*P* < 0.01 *versus* HG. (**D**) Graphic presentation of the albumin influx across podocyte monolayer. Podocyte monolayer on collagen I‐coated transwell filters was incubated with LG or HG for 48 hrs. HG resulted in impaired filtration barrier function of podocyte monolayer, which was rectified by MALAT1 siRNA transfection. Podocytes under culture of LG or transfected with cont siRNA were the controls. Values denote the mean ± S.D.; ^a^*P* < 0.01 *versus* LG,^b^*P* < 0.01 *versus* HG.

Simultaneously, high‐glucose treatment resulted in remarkable reductions in the expression of podocyte specific markers p‐cadherin and ZO‐1, as well as acquisitions of mesenchymal phenotypic marker desmin, compared with podocytes under low‐glucose conditions on both protein and mRNA levels (*P* < 0.01) (Figs 3A and B, 4A). Immunofluorescence presented that under low‐glucose conditions, p‐cadherin was highly expressed in podocytes cytoplasm and nuclei with ZO‐1 located at cell‐cell contacts, when desmin was hardly detected. After high‐glucose stimulation for 48 hrs, levels of p‐cadherin and ZO‐1 were diminished, whereas desmin expression was markedly promoted demonstrated by weakened staining of p‐cadherin and ZO‐1 but intensed staining of desmin (Figs 3C and 4B). Next, we analysed the effects of high glucose on the expression of matrix metalloproteinases (MMPs), which were responsible for the turnover and breakdown of extra‐cellular matrix (ECM) and glomerulus 34, and we found a hyperactivity of MMP‐2 was driven by high‐glucose treatment (*P* < 0.01) (Fig. 4C), along with increased albumin permeability (*P* < 0.01) (Fig. 4D).

### MALAT1 knock‐down ameliorated podocyte injury caused by high glucose

Considered that MALAT1 was boosted during the first few hours of high‐glucose stimulation, we used siRNAs to knock‐down MALAT1. Knock‐down efficiency was confirmed by real‐time PCR that showed a marked reduction in MALAT1 levels after siRNA transfection for 48 hrs (*P* < 0.01) (Fig. 2B). Intriguingly, transfection with MALAT1 siRNA plus high‐glucose stimulation for 48 hrs neither led to a greater extent of down‐regulation of MALAT1 levels (*P* > 0.05) (Fig. 2C), nor any dislocation as was shown in FISH (Fig. 2A), compared with podocytes without transfection, whereas partially reversed podocyte damage caused by high glucose: p‐cadherin and ZO‐1 expression were augmented, and desmin expression were prohibited on both protein and mRNA levels (*P* < 0.05) (Figs 3A and B, 4A). Immunofluorescence demonstrated that the staining of p‐cadherin and ZO‐1 were rectified, and desmin staining was decreased (Fig. 3C and 4B). MMP‐2 activity was also abrogated by MALAT1 siRNA transfection (*P* < 0.01) (Fig. 4C). The restoration of podocyte dysfunction was further confirmed by albumin permeability assay, which illustrated that albumin passage was significantly decreased at time‐points of 2, 4 and 6 hrs, in comparison with that under high‐glucose conditions (*P* < 0.01) (Fig. 4D). These aforementioned observations indicated that dynamic alterations of MALAT1, its up‐regulation during the first few hours in particular was indispensable in high glucose‐induced podocyte dysfunction, and early interference of MALAT1 showed a protective effect on functional integrity of podocytes under such harsh circumstances.

### SRSF1 was a MALAT1 RNA‐binding protein, overexpressed after high‐glucose stimulation but partially reversed by MALAT1 siRNA

**Figure 5:**
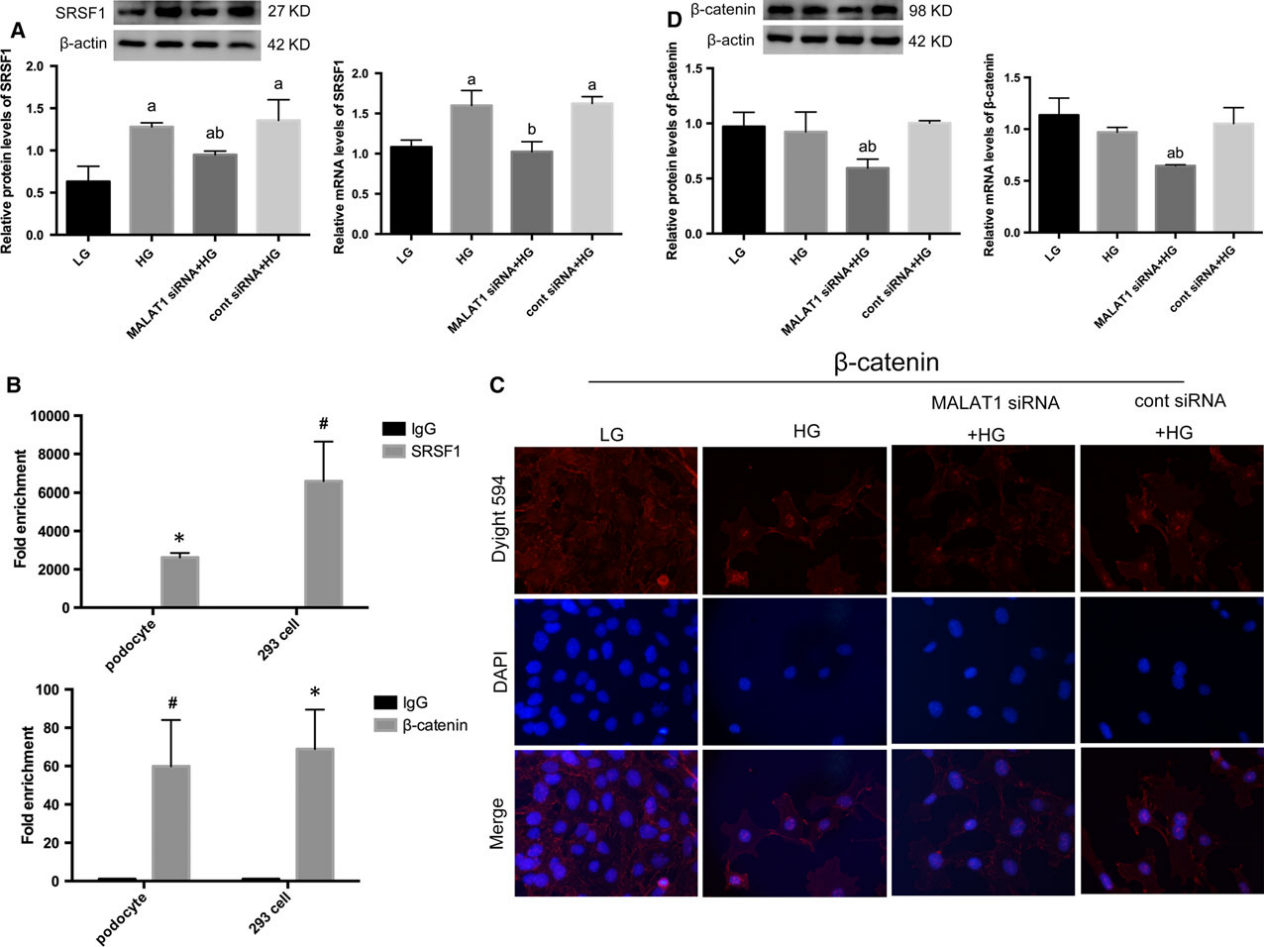
Effects of MALAT1 knock‐down on β‐catenin and SRSF1. (**A**) A 48 hrs of HG incubation was sufficient to result in an overexpression of SRSF1, compared with LG; this mutation was partially reversed by MALAT1 siRNA transfection. Podocytes under culture of LG or transfected with cont siRNA were the controls. Values denote the mean ± S.D.; ^a^*P* < 0.01 *versus* LG,^b^*P* < 0.05 *versus* HG. (**B**) RIP analysis using anti‐SRSF1 or anti‐β‐catenin antibodies were performed either in cultured mouse podocytes with or without β‐catenin shRNA transfection, or in human 293 cells. A control IgG was used as the negative control for immunoprecipitation. Real‐time PCR was used to determine the RIP signals. Values denote the mean ± S.D.; *P < 0.01 *versus* IgG, ^#^ *P* < 0.05 *versus* IgG. (**C,D**) Immunofluorescence showed β‐catenin nuclear translocation was triggered by HG stimulation, although total protein levels were not significantly altered; MALAT1 knockdown significantly down‐regulated β‐catenin expression on both protein and mRNA levels, with diminished high glucose‐induced nuclear accumulation of β‐catenin. Podocytes under culture of LG or transfected with cont siRNA were the controls. Values denote the mean ± S.D.; ^a^*P* < 0.01 *versus* LG,^b^*P* < 0.01 *versus* HG.

*In vivo*, we have shown an up‐regulation of SRSF1 mRNA levels. Next, we examined SRSF1 expression in cultured podocytes. Results showed that high glucose resulted in a notable increase in SRSF1 expression on both protein and mRNA levels (*P* < 0.01), which were significantly decreased by MALAT1 knock‐down, compared with podocytes without transfection (*P* < 0.05) (Fig. 5A), suggesting a potential interplay between MALAT1 and SRSF1 in mouse podocytes. To this end, we performed nuclear RNA immunoprecipitation assay using an anti‐SRSF1 antibody or normal mouse IgG as a control. Real‐time PCR of the former samples using MALAT1 primers showed a significant amplification or enrichment, either in cultured mouse podocytes (*P* < 0.01) or in human 293 cells (*P* < 0.05) (Fig. 5B), confirming that the interactions between MALAT1 and SRSF1 potentially depended on their mutual physical binding, and this specific binding might exist in a ubiquitous pattern.

### Nuclear translocation of β‐catenin was accompanied with the decline of MALAT1 induced by high glucose but prevented by MALAT1 knock‐down

Our previous studies have shown that dephosphorylated β‐catenin, or the active form of β‐catenin was increased after high‐glucose treatment for 48 hrs 18. Here by immunofluorescence, we showed that high‐glucose treatment led to β‐catenin nuclear translocation (Fig. 5C), leaving total β‐catenin levels unchanged (*P* > 0.05) (Fig. 5D), which occurred concomitantly with the decline of MALAT1 and abnormalities of cellular markers and function. Recent studies demonstrated that MALAT1 promoted cell migration and invasion in OSCC by triggering nuclear accumulation of NF‐κB and β‐catenin 12, illustrating a potential interplay between MALAT1 and β‐catenin. Therefore, we performed nuclear RNA immunoprecipitation and found by real‐time PCR that β‐catenin bound to MALAT1, demonstrated by a marked amplification of MALAT1 in samples using anti‐β‐catenin antibodies, compared with the control, either in mouse podocytes (*P* < 0.05) or human 293 cells (*P* < 0.01) (Fig. 5B). This interplay was further identified by Western blot analysis and real‐time PCR that β‐catenin expression on protein and mRNA levels were significantly abolished in podocytes transfected with MALAT1 siRNA, compared with podocytes treated with high glucose (*P* < 0.01) (Fig. 5D).

### β‐catenin was involved in modulation of MALAT1 transcription *via* binding to its promoter region

**Figure 6:**
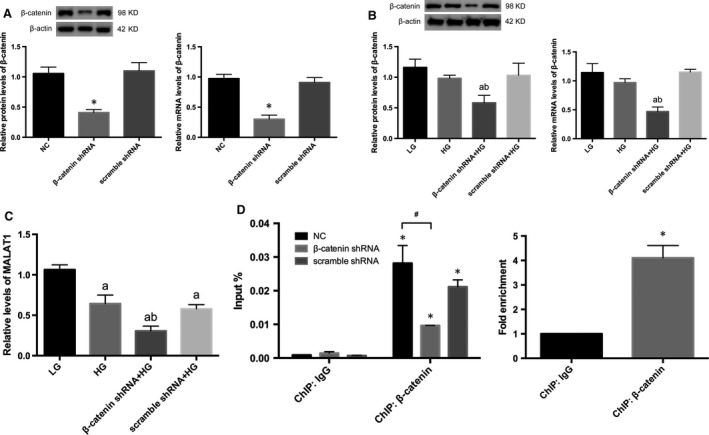
Effects of β‐catenin knock‐down on MALAT1. (**A**) β‐catenin knock‐down efficiency was determined by Western blot and real‐time PCR analysis. Results demonstrated a significant abrogation of β‐catenin expression after transduction of lentiviral shRNAs. Podocytes under normal conditions or transfected with scramble shRNA were the controls. *P<0.01 *versus* NC or scramble shRNA. (**B**) β‐catenin shRNA significantly inhibited β‐catenin expression on both protein and mRNA levels. Podocytes under culture of LG or transfected with cont siRNA were the controls. Values denote the mean ± S.D.; ^a^*P* < 0.01 *versus* LG,^b^*P* < 0.01 *versus* HG. (**C**) β‐catenin knock‐down resulted in a further degree of MALAT1 down‐regulation, in comparison with that in podocytes in exposure of HG. Podocytes under culture of LG or transfected with cont siRNA were the controls. Values denote the mean ± S.D.; ^a^*P* < 0.01 *versus* LG,^b^*P* < 0.01 *versus* HG. (**D**) ChIP analysis using anti‐β‐catenin or anti‐IgG antibodies were performed either in cultured mouse podocytes with or without β‐catenin shRNA transfection, or in human 293 cells. Real‐time PCR was used for the detection of the ChIP signals. Values denote the mean ± S.D.; *P < 0.01 *versus* IgG, ^#^ *P* < 0.01 *versus* NC.

**Figure 7:**
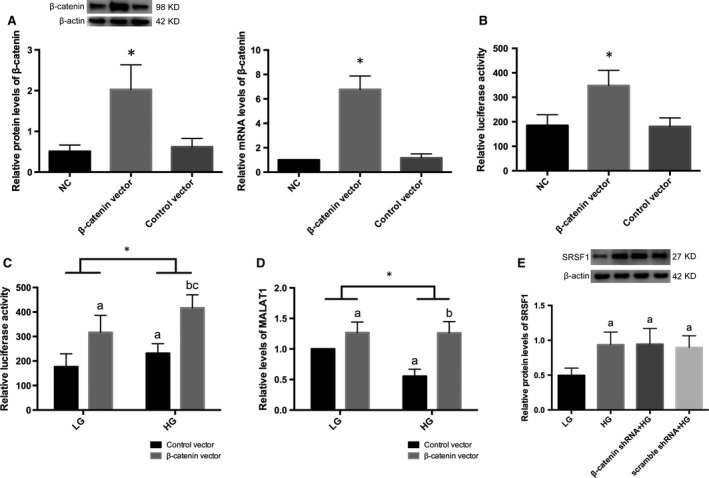
Effects of β‐catenin overexpression on MALAT1 promotor activity. (**A**) β‐catenin overexpression was examined by Western blot and real‐time PCR analysis. Podocytes under normal conditions or transfected with empty vectors were the controls. Values denote the mean ± S.D. *P<0.01 *versus* NC or control vector. (**B**) Luciferase reporter system was used to determine the MALAT1 promotor activity. Relative luciferase activity was enhanced after β‐catenin overexpression. Podocytes under normal conditions or transfected with empty vectors were the controls. Values denote the mean ± S.D. *P<0.01 *versus* NC or control vector. (**C**) Podocytes overexpressing β‐catenin or control cells were cotransfected with pGL3‐*MALAT1* promotor‐luciferase reporter and pGL3‐Renilla luciferase reporter, and were further incubated with LG or HG for 48 hrs. Relative luciferase activity was measured. Overexpression of β‐catenin resulted in enhanced luciferase activity under either LG or HG conditions; HG led to more active luciferase activity than LG in cells where β‐catenin was overexpressed; and overall luciferase activities were significantly up‐regulated in HG‐treated podocytes than LG counterparts. Values denote the mean ± S.D.; ^a^*P* < 0.05 *versus* control vector under LG,^b^*P* < 0.01 *versus* control vector under HG,^c^*P* < 0.01 *versus* β‐catenin vector under LG using one‐way anova+LSD‐*t* test, *P < 0.01 *versus* LG using two‐way anova. (**D**) MALAT1 levels were determined by real‐time PCR after cotranfection with luciferase reporter and incubation with either LG or HG for 48 hrs in mouse podocytes overexpressing β‐catenin or control podocytes. β‐catenin overexpression caused a significant upmodulation of MALAT1 levels under LG conditions, whereas showed no further promoting effects after HG treatment; MALAT1 levels were reduced in control podocytes under HG conditions; and overall mature MALAT1 levels were significantly lower in HG‐treated podocytes than LG counterparts. Values denote the mean ± S.D.; ^a^*P* < 0.05 *versus* control vector under LG,^b^*P* < 0.01 *versus* control vector under HG using one‐way anova+LSD‐*t* test, *P < 0.05 *versus* LG using two‐way anova. (**E**) Effects of β‐catenin shRNA on SRSF1 protein expression. SRSF1 protein levels were promoted after HG stimulation; β‐catenin knock‐down had limited impacts on SRSF1 protein expression, suggesting β‐catenin might serve as a downstream effector of SRSF1. Podocytes under culture of LG or transfected with cont siRNA were the controls. Values denote the mean ± S.D.; ^a^*P* < 0.05 *versus* LG.

To further illustrate the potential interactions between MALAT1 lncRNA and β‐catenin, we used β‐catenin shRNA to knock down β‐catenin expression (*P* < 0.01) (Fig. 6A). Results demonstrated β‐catenin knock‐down not only inhibited β‐catenin expression under high‐glucose conditions (*P* < 0.01) (Fig. 6 B) but also resulted in a further degree of MALAT1 down‐regulation (*P* < 0.01) (Fig. 6C), suggesting that nuclear‐translocated β‐catenin might be involved in the progression of MALAT1 expression. Thus in this study, we performed ChIP assay in cultured mouse podocytes and found that fragments of the mouse MALAT1 promoter containing nt‐710˜‐826 region were amplified by β‐catenin antibodies compared with a non‐specific IgG control (*P* < 0.01) (Fig. 6D); β‐catenin knock‐down, however, led to remarkably reduced enrichment of MALAT1 promotor fragments in ChIP samples, compared with normal podocytes (*P* < 0.01) (Fig. 6D). Similar amplifications were also observed in 293 cells by ChIP assays (*P* < 0.01) (Fig. 6D). Next, we constructed an expression vector for murine β‐catenin and a control vector. Overexpression of β‐catenin in podocytes was identified by Western blot and real‐time PCR (*P* < 0.05) (Fig. 7A). Using dual‐luciferase reporter assay, we showed that the luciferase activity was profoundly increased in podocytes overexpressing β‐catenin in comparison with the control (*P* < 0.01) (Fig. 7B). These results were in support of our hypothesis that β‐catenin regulated MALAT1 levels by interacting with MALAT1 promoter. A further incubation with low or high‐glucose treatment for 48 hrs after transfection with the reporter system revealed β‐catenin overexpression resulted in enhancement in the luciferase activity under either low‐ or high‐glucose conditions (*P* < 0.01) (Fig. 7C). Notably, the luciferase activity in high glucose‐treated podocytes, whether overexpressing β‐catenin or not, was relatively higher than counterparts incubated with low glucose (*P* < 0.01) (Fig. 7C), suggesting that nuclear‐translocated β‐catenin driven by high‐glucose stimulation, was able to augment, to some extent, the promotor activity of MALAT1. Simultaneously, real‐time PCR showed under low‐glucose conditions, β‐catenin expression vectors resulted in increased mature MALAT1 (*P* < 0.05) (Fig. 7D); levels of MALAT1 were reduced in high glucose‐treated control cells, compared with low‐glucose counterparts (*P* < 0.01) (Fig. 7D); in cells overexpressing β‐catenin, high glucose did not lead to any further up‐regulation of MALAT1 than low‐glucose controls (*P* > 0.05) (Fig. 7D); and overall levels of MALAT1 in high glucose‐stimulated cells were significantly lower than those incubated with low glucose (*P* < 0.05) (Fig. 7D), implying the presence of relatively lower levels of mature MALAT1 despite higher transcriptional levels under high‐glucose conditions than low‐glucose counterparts. In addition, we demonstrated by Western blot that high glucose‐induced SRSF1 overexpression was not significantly altered by β‐catenin gene knock‐down (*P* > 0.05) (Fig. 7E), indicating β‐catenin might serve as a downstream effector of SRSF1.

## Discussion

MALAT1 is a highly conserved mRNA‐like long non‐coding RNA (lncRNA), originally considered as a pivotal prognostic factor in various metastatic carcinomas 7, 8, 11. In the present study, we showed MALAT1 was boosted in the kidney of STZ‐induced diabetic mice, concomitantly with mutations of podocyte specific hallmarks (decreased expression of p‐cadherin and ZO‐1, and enhanced desmin expression) and marked proteinuria. Also we demonstrated an early up‐regulation of MALAT1 levels in exposure to high glucose in cultured mouse podocytes and a later down‐regulation of MALAT1, accompanied by β‐catenin nuclear translocation when podocytes were jeopardized, with increased MMP‐2 activity and albumin permeability, which was attenuated by MALAT1 knock‐down. Mounting evidence has elaborated that modifications in podocyte architecture, such as cytoskeleton alterations, disruptions of cell‐cell contacts and cell‐matrix interaction are critical events for the development and progression of glomerular proteinuria 35, 36, 37. P‐cadherin and ZO‐1 are both critical components of podocyte slit diaphragm (SD), involved in the determination of SD structure and maintenance of its property as a size and shape selective glomerular barrier 31, 38. P‐cadherin suppression by ILK activation is linked to podocyte detachment from the glomerular basement membrane (GBM) 35. The intense expression of ZO‐1 is a prerequisite for the formation of SD and allows the proper binding of SD proteins to actin cytoskeleton 39, 40. Matrix metalloproteinase‐2 (MMP‐2) contributes to GBM turnover *via* its ability to breakdown of extra‐cellular matrix (ECM) and glomerulus 41; overexpression of MMP‐2 in transgenic mice promotes renal fibrosis and generates broad spectrum of pathological and functional characteristics of human CKD 34. Therefore, our *in vivo* and *in vitro* data provided evidence that MALAT1 dysregulation played a key role in diabetic podocyte injury and proteinuria; imbalanced MALAT1 levels contributed to disruptions of the integrity of podocyte architecture and function; preserved SD structure and thus alleviated actin interference might be one of the major consequences podocytes benefited from MALAT1 deletion.

Of interest, we showed that MALAT1 knock‐down was able to reduce the expression of total protein and mRNA levels of β‐catenin. Under normal conditions MALAT1 was highly expressed and retained in the cell nucleus of podocytes. Such localization is suggestive of a potential role in the organization or regulation of gene expression 42. It has been indicated MALAT1 become enriched in nuclear speckles, when RNA polymerase II‐dependent transcription was active 21. Nuclear speckles were dynamic and irregularly shaped nuclear domains involved in pre‐mRNA splicing processing and RNA transport in mammalian cells 20. Recent evidence elaborated that MALAT1 could interact with nascent pre‐mRNAs to guide its regulatory functions 43. Thus, we inferred that β‐catenin expression was modulated at post‐transcriptional level by MALAT1, potentially *via* recognition of β‐catenin pre‐mRNAs, and thereby altered at protein levels. Intriguingly, MALAT1 levels were affected by β‐catenin as well through binding to the promotor region of MALAT1. β‐catenin shRNA transduction led to decreased mutual binding and thus diminished MALAT1 expression; overexpression of β‐catenin, on the contrary, was able to increase promotor activity of MALAT1. These observations suggested that there might exist a feedback loop between MALAT1 and β‐catenin in the podocyte nucleus under normal conditions: β‐catenin regulated MALAT1 transcription *via* binding to its promotor and regulating the promotor activity; MALAT1 in turn changed the pattern of pre‐mRNA processing of β‐catenin post‐transcriptionally. This loop was damaged by high‐glucose treatment, which resulted in an early increase in MALAT1 that contributed to nuclear accumulation of β‐catenin at later stage.

It was notable that in our observations the nuclear‐translocated β‐catenin did not significantly increase MALAT1 levels despite its binding ability to MALAT1 promotor and its enhancement in MALAT1 promotor activity, even in cells overexpressing β‐catenin, where MALAT1 levels were not significantly higher than but only comparable to low‐glucose counterparts, suggesting that MALAT1 might be as well under the control of some other molecules post‐transcriptionally after high‐glucose stimulation. Many ncRNAs were implicated in regulating RNA processing either directly through base pairing or indirectly through protein intermediates 43, 44. The interactions between MALAT1 and pre‐mRNAs were indirect and required its physical associations with SR splicing proteins 43. Here, we found in mouse podocytes that SRSF1 bound to MALAT1 in the cell nuclei. SRSF1 is the archetype member of the SR protein family, involved in key steps of mRNA metabolism, such as nuclear export of the mature mRNA 22, 45. Depletion of MALAT1 or overexpression of an SR protein could change the alternative splicing of a similar set of endogenous pre‐mRNAs 26. In concert with these studies, we showed SRSF1 expression was promoted in damaged podocytes under high‐glucose conditions after a transient increase of MALAT1, and co‐existed with the later decline of MALAT1; MALAT1 deletion reversed SRSF1 levels and podocyte damage, nevertheless, it did not lead to a further decrease in MALAT1, indicating that early augment of MALAT1, through its binding capability to SRSF1 protein, might alter the setting of alternative splicing of pre‐mRNAs of SRSF1, thus lead to enhanced mRNA levels and modified protein levels. As SRSF1 was engaged in the nonsense‐mediated mRNA decay (NMD) mRNA quality surveillance, besides its splicing capability 23, 46, we speculated early imbalance of MALAT1 might have also triggered a degradation mechanism, which, with SRSF1 potentially involved, resulted in an overcorrection of MALAT1 levels and podocyte damage under high‐glucose conditions, but prevented by MALAT1 siRNA transfection. Overall, these aforementioned data underlined a key step of early up‐regulation involved in high glucose‐induced podocyte damage.

Our *in vivo* data further identified a concomitant elevation of SRSF1 mRNA and MALAT1 in the kidney of STZ‐induced diabetic mice, suggesting MALAT1 and SRSF1, with associated disordered pre‐mRNA processing, contributed to the development and progression of diabetic nephropathy. Prior studies suggested that lncRNA MALAT1 was raised in the retinas of STZ‐induced diabetic rats, blockade of which alleviated retinal inflammation *via* reducing expression of ICAM, VEGF and TNF‐α 9; misregulation of VEGF was often observed in clinical or experimental diabetic nephropathy, involved in matrix accumulation and haemodynamic changes 28, 47. In WT‐1 mutant podocytes, SRSF1 promoted the expression of pro‐angiogenic VEGF‐a splice isoforms and caused imbalanced angiogenesis 27. Therefore, it would be intriguing to further determine whether these inflammatory cytokines were downstream targets of MALAT1 or SRSF1 in diabetic podocyte injury and related proteinuria.

Next, we were interested to find *in vitro* that β‐catenin knock‐down resulted in a further extent of MALAT1 down‐regulation, whereas SRSF1 protein expression was not significantly altered, suggesting that β‐catenin might serve as a downstream effector of SRSF1 after high‐glucose treatment. By shuttling between the nucleus and the cytoplasm, SRSF1 interacted with and activated mTOR to promote translation 22, 23, 48; dysregulation of the latter was sufficient to facilitate glomerular diseases 49. Later studies suggested that human SRSF1 and SRSF9 were responsible for the promotion of Wnt1/β‐catenin‐induced reporter expression 24. In small cell lung cancer, SRSF1 silencing suppressed PI3K/AKT and MEK/ERK pathways, activation of the latter was associated with PKB‐mediated phosphorylation of GSK‐3β thereby released and stabilized cytoplasmic β‐catenin 50, 51. In podocytes, activated PI3K/AKT pathway was involved in transcriptional suppression of nephrin, a key SD protein that anchored the SD to the actin cytoskeleton 39, 52. Thus, we concluded that MALAT1/β‐catenin loop was wrecked by high glucose with SRSF1 serving as an important mediator, which on one hand contributed to β‐catenin nuclear accumulation and down‐regulated MALAT1 on the other hand. These abnormalities were rectified by MALAT1 knock‐down *via* preventing early up‐regulation of MALAT1, that was, the initial key event in the challenge of high glucose.

Growing evidence has illustrated that MALAT1 played a negligible role in epithelial‐mesenchymal transition (EMT) of diverse cancer cells 12. Li *et al*. and our previous studies illustrated that EMT was a potential mechanism leading to podocyte malfunction 18, 53. EMT is a reversible dedifferentiation process that converts epithelial cancer cells into dedifferentiated cells, characterized by the loss of epithelial traits such as E‐cadherin and the acquisition of fibroblastic markers α‐smooth muscle actin (α‐SMA) and fibroblast‐specific protein‐1 (FSP‐1) 18, 54. The release of β‐catenin, triggered by the loss of cadherin‐mediated cell adhesion, has been extensively indicated in the literature to serve as a pivotal modulator engaged in EMT in numerous cancer cells 17, 55. Here, albeit the partial reversal of podocyte damage markers and obliterated β‐catenin nuclear accumulation, we did not observe any significant impacts of MALAT1 knock‐down on expression of critical EMT hallmarks such as α‐SMA, FSP‐1 or Snail (data not shown), hence whether MALAT1 had a major contribution to high glucose‐associated podocyte EMT was still debated and undefined, which should be attached importance to in future investigations.

**Figure 8:**
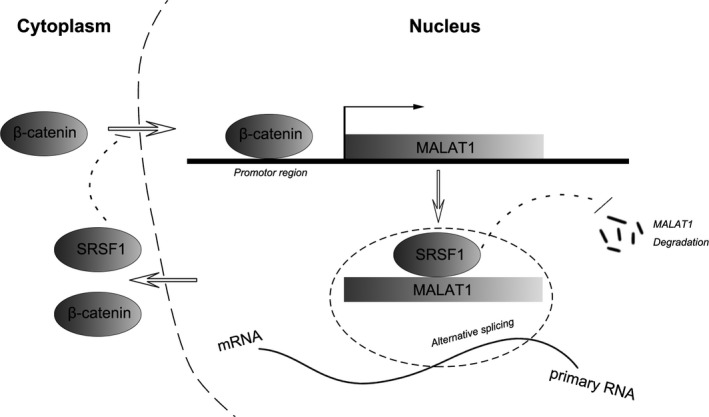
Schematic drawing depicting the feedback regulation between MALAT1 and β‐catenin in mouse podocytes under culture, with SRSF1 serving as a pivotal mediator.

In summary, we demonstrated that MALAT1 misregulation was involved in the progression of diabetic nephropathy, and we showed a potential feedback loop between MALAT1 and β‐catenin playing a role in high glucose‐associated podocyte impairment, with SRSF1 as a important participant potentially *via* changing the pattern of alternative splicing of gene targets through its physical binding to MALAT1; inhibition of MALAT1 broke the cascade and rectified podocytes dysfunction stimulated by high glucose (Fig. 8). These observations provided evidence for an indispensable role of lncRNA MALAT1 in diabetic nephropathy and high glucose‐associated podocyte damage, and a further understanding of lncRNAs in biological processes in the kidney.

## Conflicts of interest

The authors declare that there is no conflict of interest associated with this manuscript.
